# Radiographic indices for lumbar developmental spinal stenosis

**DOI:** 10.1186/s13013-017-0113-3

**Published:** 2017-02-20

**Authors:** Jason Pui Yin Cheung, Karen Ka Man Ng, Prudence Wing Hang Cheung, Dino Samartzis, Kenneth Man Chee Cheung

**Affiliations:** Department of Orthopaedics and Traumatology, Queen Mary Hospital, The University of Hong Kong, Pokfulam Road, Hong Kong, SAR China

**Keywords:** Developmental spinal stenosis, Radiological indices, MRI, X-ray

## Abstract

**Background:**

Patients with developmental spinal stenosis (DSS) are susceptible to developing symptomatic stenosis due to pre-existing narrowed spinal canals. DSS has been previously defined by MRI via the axial anteroposterior (AP) bony spinal canal diameter. However, MRI is hardly a cost-efficient tool for screening patients. X-rays are superior due to its availability and cost, but currently, there is no definition of DSS based on plain radiographs. Thus, the aim of this study is to develop radiographic indices for diagnosing DSS.

**Methods:**

This was a prospective cohort of 148 subjects consisting of patients undergoing surgery for lumbar spinal stenosis (patient group) and asymptomatic subjects recruited openly from the general population (control group). Ethics approval was obtained from the local institutional review board. All subjects underwent MRI for diagnosing DSS and radiographs for measuring parameters used for creating the indices. All measurements were performed by two independent investigators, blinded to patient details. Intra- and interobserver reliability analyses were conducted, and only parameters with near perfect intraclass correlation underwent receiver operating characteristic (ROC) analysis to determine the cutoff values for diagnosing DSS using radiographs.

**Results:**

Imaging parameters from a total of 66 subjects from the patient group and 82 asymptomatic subjects in the control group were used for analysis. ROC analysis suggested sagittal vertebral body width to pedicle width ratio (SBW:PW) as having the strongest sensitivity and specificity for diagnosing DSS. Cutoff indices for SBW:PW were level-specific: L1 (2.0), L2 (2.0), L3 (2.2), L4 (2.2), L5 (2.5), and S1 (2.8).

**Conclusions:**

This is the first study to define DSS on plain radiographs based on comparisons between a clinically relevant patient group and a control group. Individuals with DSS can be identified by a simple radiograph using a screening tool allowing for better cost-saving means for clinical diagnosis or research purposes.

## Background

Lumbar spinal stenosis is a constriction of the spinal canal that can cause compression of the neural tissue. Patients can experience symptoms of leg pain, radiculopathy, and claudication [1]. The cause of lumbar spinal stenosis can be grossly classified as developmental, degenerative, or a combination of both [2–5]. The degree of constriction required to cause symptoms is unclear, but with a developmentally narrowed spinal canal, patients are more susceptible to canal compression.

Lumbar developmental spinal stenosis (DSS) is likely a result of abnormal fetal and postnatal development of the lumbar vertebrae [6–8]. The definition of developmental narrowing has been suggested by Verbiest [7] to be an abnormally short anteroposterior (AP) canal diameter. The proposed absolute value of less than 10 mm is commonly accepted as canal narrowing [5, 8], but the method for coming up with this value is based on intraoperative measurements in a small number of operated cases and hence cannot be directly translated to imaging. In addition, magnification errors are common for radiographs, and these measurements should be standardized to other parameters such as an individual’s vertebral body size [9]. Other imaging-based criteria have been suggested in the past [7, 8, 10–17] but were based on inconsistent imaging modalities [8, 10, 13, 16, 17], heterogeneous populations [8, 10, 11, 13, 16, 18, 19], lacked control groups [8, 10, 11, 13, 19], and generalized measurements of the entire lumbar spine [8, 10, 11, 13, 16–19].

Cheung et al. [2] previously defined the lumbar DSS phenotype in a large-scale homogenous group of southern Chinese with standardized measurements based on magnetic resonance imaging (MRI). The axial AP bony spinal canal diameter translated to the pedicle width and generally decreased from cranially to caudally. Its cutoff values were defined using data derived from both symptomatic and asymptomatic subjects with high sensitivity and specificity values. The results from this study suggest that DSS plays an important role in the pathogenesis of symptomatic lumbar spinal stenosis. However, no similar study has been conducted on plain radiographs.

MRI is the gold standard for the assessment of patients with spinal stenosis. As a diagnostic imaging tool, it has no equal in assessment of intervertebral disc abnormalities and canal stenosis [20, 21]. Despite the advantages of using MRI for the diagnosis of lumbar DSS, there are cost concerns for overuse. If MRI is used in all suspected cases of spinal stenosis for either clinical management or research, the financial burden is astronomical. Therefore, MRI is not a cost-efficient tool for screening patients for lumbar DSS. Alternatively, plain radiographs are superior for screening due to low cost and availability.

**Fig. 1:**
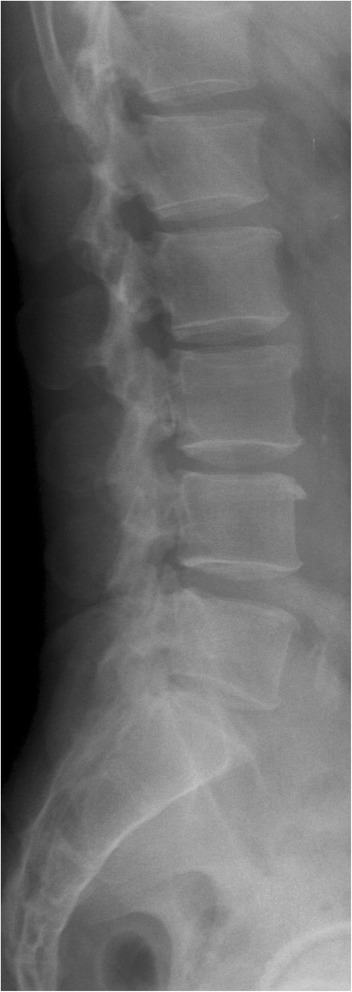
Example of a developmentally narrowed spinal canal depicted by short pedicles

**Fig. 2:**
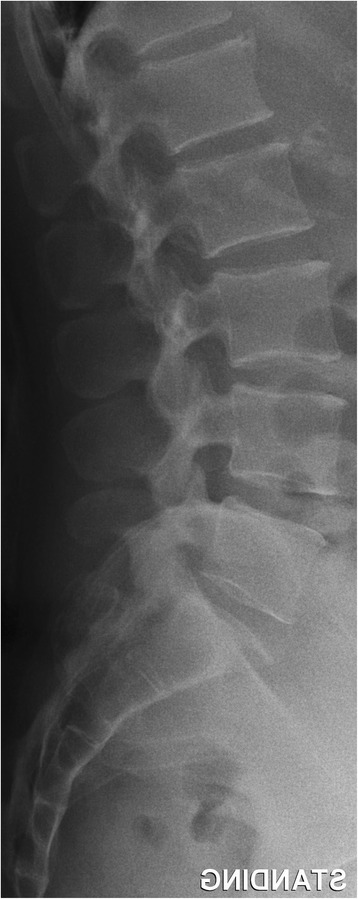
Example of a normal sized spinal canal

In the eyes of experienced clinicians, radiographs with short pedicles suggestive of DSS may be identified (Figs. 1 and 2). Several studies [22–25] have discussed canal narrowing and its measurements in the past, but these analyses were not based on a derived radiographic index and thus are subject to influence by body size. In addition, it is difficult to determine from a simple visual inspection whether pedicles are short or not because pedicle widths reduce from cranial to caudally. An attempt in creating radiographic indices has been performed in the past [26], but this was based on the comparison of MRI dural sac diameters which is affected by degenerative changes and cannot be contributed to developmental malformation. Moreover, no description has been made regarding how radiographic measurements were performed limiting relevance of their findings to actual developmental narrowing of the bony spinal canal. Therefore, there is a need for an easily used radiographic definition for lumbar DSS. As such, the aim of this study is to develop practical radiographic indices for diagnosing DSS.

## Methods

### Study design and population

This was a prospectively collected cohort of 66 patients who underwent surgery for lumbar spinal stenosis (patient group) and 82 asymptomatic subjects who were openly recruited from the general population via advertisement (control group) as part of the Hong Kong Disc Degeneration Cohort study [27–30]. There were 34 females (51.5%) and 32 males (48.5%) in the patient group with mean age of 65.9 years (±SD 10.9). There were 31 males (38.3%) and 50 females (61.7%) in the control group with mean age of 56.4 years (±SD 6.8). Ethics review was performed by a local institutional review board. All subjects were of Chinese ethnicity and were recruited via written consent since December 2012. Subject recruitment ended on December 2014. Subjects with congenital deformities, previous infections, tumors, trauma, or spondylolisthesis were excluded from the study. Various patient demographics and clinical profile were noted, including age and sex and, for the patient group, symptomatology, operation performed, and number of operated levels. All subjects underwent MRI and standing AP and lateral radiographs of the lumbosacral spine. For the patient group, all imaging were performed preoperatively.

### MRI measurements

**Fig. 3:**
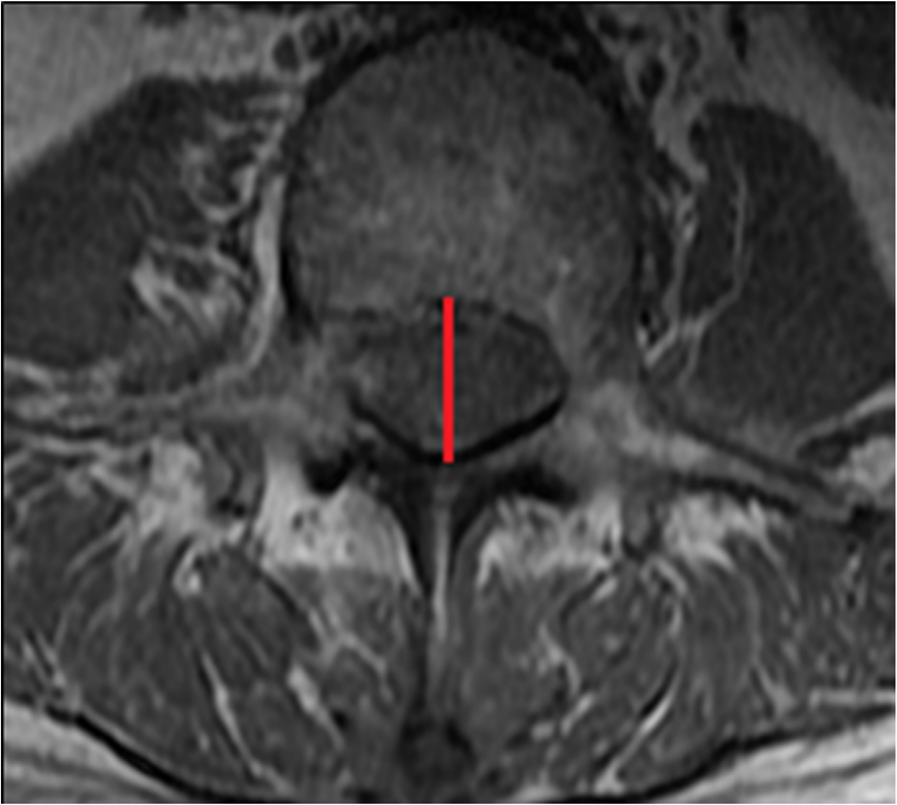
Axial T1 MRI image showing the measurement for the anteroposterior bony spinal canal diameter

Axial T1-weighted MRI images of the lumbar spine from L1 to S1 were utilized for all subjects. 1.5 or 3 T HD MRI machines were used for imaging. The field of view was 18 × 18 cm, slice thickness was 4 mm, and slice spacing was 0 mm. The imaging matrix was 288 × 192. The repetition time (TR) was 700–800 ms, and the echo time (TE) was 8–10 ms for the T1 images. There were 11 slices per vertebral level, and parallel slices were made according to the disc and pedicle levels. The axial image used for measurement was the cut with the thickest pedicle diameter and could also visualize the whole bony ring at the pedicle level. The midline AP bony spinal canal diameter was used to diagnose DSS (L1 <20 mm, L2 <19 mm, L3 <19 mm, L4 <17 mm, L5 <16 mm, S1 <16 mm) [2, 31]. Only the AP bony spinal canal diameter (Fig. 3) was used because it was most representative of DSS. The subjects in the control group were all confirmed to have normal sized spinal canals by the MRI cutoff values discussed.

### Plain radiographic assessment

**Fig. 4:**
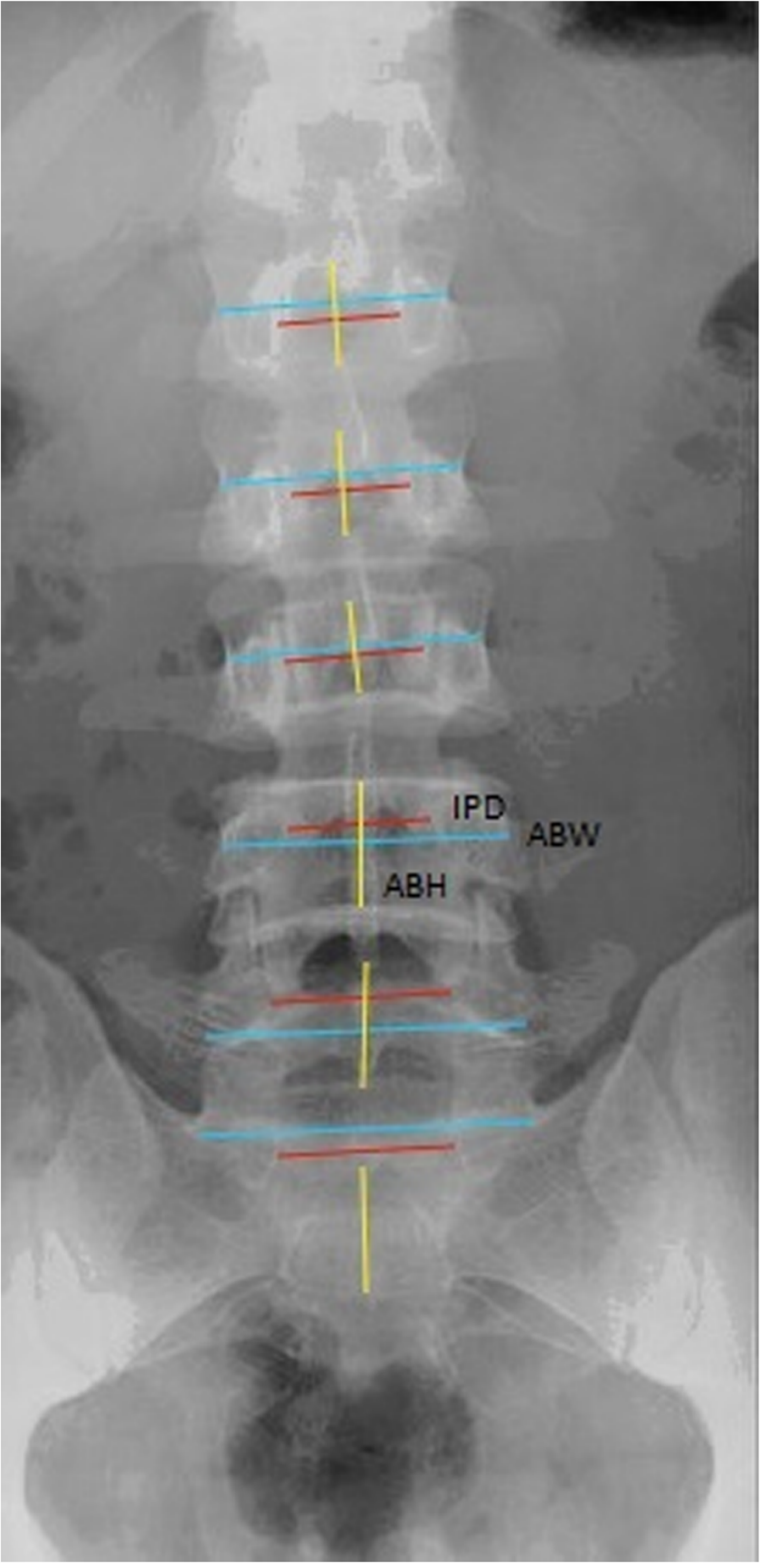
Measurement scheme for the anteroposterior standing radiograph: axial vertebral body width (*ABW: light blue*), axial vertebral body height (*ABH: yellow*), and interpedicular distance (*IPD: red*)

**Fig. 5:**
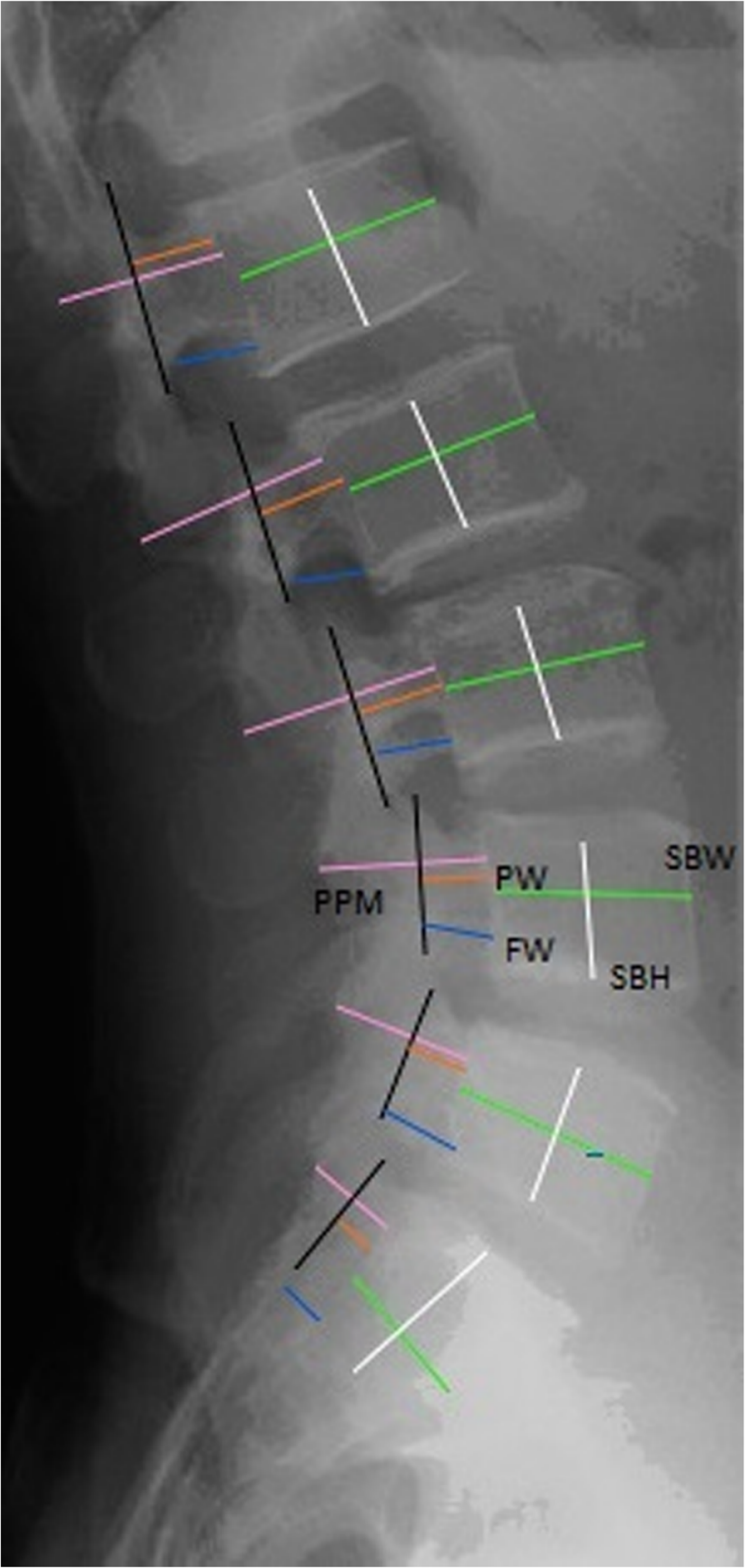
Measurement scheme for the lateral standing radiograph: sagittal vertebral body width (*SBW: green*), sagittal vertebral body height (*SBH: white*), pedicle width (*PW: orange*), posterior pedicle margin (*PPM: pink*), and foraminal width (*FW: dark blue*). The *black line* indicates how the line connecting the facet joints should be outlined to identify the posterior margin of the pedicle width

All subjects underwent lumbar AP and lateral standing radiographs of the lumbosacral spine (view of the thoracolumbar region to sacrum) extracted to measure parameters including interpedicular distance (IPD) and axial vertebral body height and width (ABW) on AP views (Fig. 4) and foraminal width (FW), pedicle width (PW), posterior pedicle margin (PPM), and sagittal vertebral body height and width (SBW) on lateral views (Fig. 5). The FW was taken at the widest diameter below the pedicle and above the intervertebral disc. The PW was measured from the posterior border of the vertebral body to the line connecting the cranial and caudal facet joints. The PPM was measured from the posterior vertebral body to the base of the spinous process. These were the most consistent landmarks visible on lateral radiographs. The IPD on the AP view was taken at the narrowest horizontal diameter between the two pedicles. The vertebral body height and width measurements were taken at the midpoint of the vertebral body in both AP and lateral radiographs from the superior endplate to the inferior endplate. In case of any film rotation, there will be a “double feature” of the landmarks. For these cases, the midpoint between the more proximal and more distal landmarks was taken as the correct measurement point.

### Image analysis

All measurements were performed independently by two investigators, and all clinical information was blinded to the investigators during measurements. For reliability testing, 20 subjects were randomly selected from both groups for intra- and interobserver reliability assessments. The first and second round of measurements was performed at least 1 month apart. Radiographs and MRIs were measured separately and not consecutively for any single subject to avoid bias during measurements. The blinding and reliability procedures were arranged by a third independent investigator who performed scrambling of the images and order of subjects prior to the measurements. All images were measured using the Centricity Enterprise Web V3.0 (GE Medical Systems, 2006).

### Statistical analysis

Descriptive and frequency statistics were performed of the data. Median values were used for analysis of the different parameters and ratios to avoid skewing of the data. Reliability assessment was based on intraclass correlation (ICC) analysis. ICC could be interpreted based on the following alpha values: 0–0.29 indicated poor agreement, 0.30–0.49 indicated fair agreement, 0.50–0.69 indicated moderate agreement, 0.70–0.80 indicated strong agreement, and >0.80 indicated almost perfect agreement [32, 33]. The 95% confidence interval (CI) bounds were assessed for precision. A *p* value of <0.05 was considered significant. Only radiographic parameters with near-perfect agreement were used for radiographic indices and underwent receiver operating characteristic (ROC) analysis to identify the cutoff values that diagnose subjects with DSS. Cutoff values with the highest sensitivity and specificity results were chosen.

## Results

The MRI diameters and reliability assessment of both groups were listed in Table 1. The median AP bony spinal canal diameters of the patient group gradually decreased from cranial to caudally while the normal subjects were generally similar throughout the levels. According to the criteria for diagnosing DSS on MRI, all 66 subjects in the patient group had DSS while none of the 82 asymptomatic subjects had developmental canal narrowing. Both intra- and interobserver reliability for the AP bony spinal canal diameter on MRI were near perfect using ICC analysis. Only clinical symptomatic levels from L3 to S1 were observed in the patient group.

**Table 1:** MRI measurements of lumbar developmental spinal stenosis

Measurement	Median, mm (±SD)		Intraobserver reliability	95% CI	Interobserver reliability	95% CI
AP bony spinal canal diameter	Patient	Control				
L1	18.2 (1.4)	19.3 (2.3)	0.94*	0.75–0.99	0.91*	0.70–0.97
L2	17.3 (1.5)	19.1 (2.3)	0.97*	0.87–0.99	0.96*	0.87–0.98
L3	16.5 (2.0)	18.8 (2.9)	0.98*	0.94–1.00	0.97*	0.88–0.99
L4	14.8 (1.9)	19.1 (2.1)	0.97*	0.89–0.99	0.94*	0.82–0.99
L5	14.2 (1.3)	19.4 (3.8)	0.98*	0.91–0.99	0.99*	0.87–0.99
S1	14.1 (1.6)	17.4 (2.1)	0.96*	0.82–0.99	0.89*	0.78–0.99

*Abbreviations*: *SD* standard deviation, *AP* anteroposterior, *CI* confidence interval

*p < 0.001

Almost perfect ICC agreement was found for PW, PPM, SBW, ABW, and IPD (Table 2). The PW and PPM measurements gradually decreased from cranial to caudally for the patient group, but this trend only existed for PW in the control group. The ABW and IPD gradually increased from cranial to caudally for both groups. According to the ICC agreement, three radiographic indices were created (two from lateral radiographs and one from AP radiographs). For the lateral radiograph, SBW:PW and SBW:PPM ratios were calculated. Similarly, ABW:IPD was calculated for the AP radiograph (Table 3).

**Table 2:** Radiograph measurements and reliability analysis

Measurement	Median, mm (±SD)		Interobserver reliability	95% CI^*^	Intraobserver reliability	95% CI^*^
	Patient	Control				
Foraminal width						
L1	11.8 (1.8)	13.1 (1.5)	0.84	0.59–0.94	0.96	0.91–0.99
L2	11.1 (1.6)	13.2 (1.8)	0.78	0.43–0.92	0.89	0.71–0.96
L3	10.5 (1.9)	12.8 (1.6)	0.86	0.64–0.95	0.93	0.83–0.97
L4	9.5 (1.9)	11.1 (1.7)	0.84	0.58–0.94	0.89	0.70–0.96
L5	8.0 (1.9)	9.3 (1.6)	0.72	0.27–0.89	0.88	0.70–0.96
S1	6.6 (2.0)	7.5 (1.5)	0.92	0.55–0.99	0.86	0.63–0.95
Pedicle width						
L1	16.5 (2.5)	17.9 (2.2)	0.95	0.87–0.98	0.95	0.88–0.98
L2	16.7 (2.5)	17.7 (1.8)	0.97	0.93–0.99	0.91	0.78–0.97
L3	15.0 (1.9)	18.3 (1.8)	0.96	0.90–0.99	0.89	0.72–0.96
L4	14.6 (2.8)	17.4 (2.1)	0.97	0.92–0.99	0.90	0.75–0.96
L5	11.3 (1.9)	16.0 (2.5)	0.95	0.86–0.98	0.95	0.88–0.98
S1	8.2 (2.3)	11.2 (3.8)	0.98	0.93–0.99	0.93	0.81–0.97
Posterior pedicle margin						
L1	27.6 (3.8)	28.0 (3.1)	0.97	0.93–0.99	0.98	0.95–0.99
L2	28.5 (4.0)	27.7 (2.6)	0.99	0.96–0.99	0.97	0.91–0.99
L3	29.7 (3.5)	29.3 (2.4)	0.98	0.94–0.99	0.93	0.81–0.97
L4	28.5 (4.4)	29.7 (2.7)	0.97	0.93–0.99	0.93	0.81–0.97
L5	25.6 (4.4)	27.5 (2.9)	1.00	0.99–1.00	0.97	0.93–0.99
S1	20.6 (4.2)	21.9 (4.0)	0.99	0.98–1.00	0.95	0.86–0.98
Sagittal vertebral body width						
L1	35.6 (4.7)	35.0 (3.9)	0.97	0.92–0.99	0.94	0.85–0.98
L2	37.3 (5.3)	35.8 (4.3)	0.97	0.91–0.99	0.95	0.87–0.98
L3	39.3 (4.6)	37.2 (4.0)	0.97	0.91–0.99	0.96	0.90–0.99
L4	39.7 (3.9)	36.5 (3.9)	0.95	0.86–0.98	0.93	0.82–0.97
L5	39.3 (3.8)	36.5 (3.7)	0.97	0.92–0.99	0.96	0.90–0.99
S1	27.9 (4.0)	29.4 (3.9)	0.91	0.77–0.97	0.90	0.75–0.96
Sagittal vertebral body height						
L1	29.0 (3.4)	28.6 (3.6)	0.97	0.91–0.99	0.94	0.83–0.98
L2	29.6 (3.4)	29.9 (2.4)	0.96	0.90–0.99	0.87	0.67–0.95
L3	29.3 (3.3)	30.2 (2.5)	0.90	0.75–0.96	0.62	0.01–0.85
L4	28.3 (3.7)	30.3 (2.3)	0.95	0.88–0.98	0.94	0.84–0.98
L5	27.6 (3.9)	30.0 (2.5)	0.93	0.81–0.97	0.94	0.85–0.98
S1	31.6 (3.5)	32.2 (3.6)	0.82	0.53–0.93	0.98	0.94–0.99
Interpedicular distance						
L1	24.5 (2.3)	25.9 (2.7)	0.94	0.84–0.97	0.96	0.89–0.98
L2	24.8 (2.2)	26.2 (2.6)	0.92	0.79–0.97	0.96	0.91–0.99
L3	26.2 (3.0)	27.7 (2.1)	0.97	0.92–0.99	0.98	0.95–0.99
L4	27.7 (4.0)	29.7 (2.7)	0.97	0.92–0.99	0.98	0.94–0.99
L5	30.7 (4.2)	34.2 (3.3)	1.00	0.99–1.00	0.95	0.88–0.98
S1	34.4 (4.9)	37.5 (3.2)	0.99	0.96–1.00	0.94	0.84–0.98
Axial vertebral body width						
L1	43.0 (4.2)	41.6 (4.3)	0.98	0.96–0.99	1.00	0.99–1.00
L2	45.7 (4.3)	42.2 (4.6)	0.98	0.96–0.99	0.99	0.98–1.00
L3	47.1 (4.5)	44.1 (4.8)	0.99	0.97–1.00	0.98	0.94–0.99
L4	50.6 (5.0)	48.2 (4.9)	0.98	0.96–0.99	0.94	0.84–0.98
L5	53.7 (5.9)	55.4 (5.7)	0.98	0.95–0.99	0.94	0.84–0.98
Axial vertebral body height						
L1	25.3 (4.1)	29.1 (3.1)	0.98	0.96–0.99	0.68	0.19–0.87
L2	27.0 (3.9)	29.9 (2.8)	0.98	0.95–0.99	0.98	0.95–0.99
L3	27.2 (3.3)	30.0 (2.4)	0.95	0.87–0.98	0.93	0.81–0.97
L4	26.7 (3.6)	30.6 (2.7)	0.92	0.80–0.97	0.82	0.55–0.93
L5	28.1 (4.4)	28.1 (4.3)	0.95	0.86–0.98	0.78	0.43–0.91

*Abbreviations*: *SD* standard deviation, *CI* confidence interval

^*^Statistical significance (all *p* values <0.05)

**Table 3:** Radiographic indices for lumbar developmental spinal stenosis

Measurement	Median (±SD)		Median (±SD)		Median (±SD)	
	SBW:PW		SBW:PPM		ABW:IPD	
	Patient	Control	Patient	Control	Patient	Control
L1	2.2 (0.4)	2.0 (0.4)	1.3 (0.2)	1.2 (0.2)	1.8 (0.2)	1.6 (0.2)
L2	2.4 (0.5)	2.0 (0.3)	1.3 (0.3)	1.3 (0.2)	1.8 (0.2)	1.6 (0.2)
L3	2.6 (0.5)	2.0 (0.2)	1.4 (0.2)	1.2 (0.2)	1.8 (0.2)	1.6 (0.2)
L4	2.8 (0.8)	2.1 (0.3)	1.4 (0.4)	1.2 (0.1)	1.8 (0.3)	1.6 (0.2)
L5	3.5 (1.4)	2.3 (0.4)	1.6 (0.3)	1.3 (0.2)	1.7 (0.2)	1.6 (0.2)
S1	3.5 (1.7)	2.8 (0.9)	1.4 (0.4)	1.4 (0.2)	1.8 (0.4)	1.5 (0.1)

*Abbreviations*: *SD* standard deviation, *SBW* sagittal vertebral body width, *PW* pedicle width, *PPM* posterior pedicle margin, *ABW* axial vertebral body width, *IPD* interpedicular distance

ROC analysis (Table 4) suggested that the SBW:PW ratio had the highest area under the curve analysis and strongest sensitivity and specificity results. In addition, the overall median values for SBW:PW had a wider difference in margin value between patient and control groups while the indices for SBW:PPM and ABW:IPD did not have a significant difference between groups to represent a clinically useful cutoff value. For SBW:PW, level-specific cutoff values were suggested: L1 (2.0), L2 (2.0), L3 (2.2), L4 (2.2), L5 (2.5), and S1 (2.8). As a simple guideline, developmental canal narrowing could be defined as an index greater than 2.8 for SBW:PW. This was a general statement of the calculated results using the largest index (S1) for SBW:PW. This was an attempt to avoid over-diagnosis of DSS since the indices were level-specific and some of the lumbosacral levels had smaller indices than others.

**Table 4:** Cutoffs for lumbar developmental spinal stenosis

	Cutoff	Sensitivity	Specificity	Area under curve	*p* value	95% CI
SBW:PW						
L1	2.0	0.76	0.50	0.67	0.18	0.53–0.81
L2	2.0	0.78	0.67	0.73	0.06	0.58–0.89
L3	2.2	0.90	0.83	0.92	0.001	0.83–1.00
L4	2.2	0.92	0.83	0.94	<0.001	0.88–1.00
L5	2.5	0.90	0.99	0.96	<0.001	0.91–1.00
S1	2.8	0.81	0.99	0.91	0.001	0.84–0.99
SBW:PPM						
L1	1.2	0.64	0.50	0.57	0.56	0.42–0.73
L2	1.2	0.68	0.67	0.58	0.54	0.36–0.79
L3	1.2	0.76	0.67	0.66	0.20	0.45–0.87
L4	1.3	0.70	0.83	0.77	0.03	0.64–0.91
L5	1.4	0.71	0.83	0.81	0.01	0.68–0.94
S1	1.4	0.56	0.67	0.58	0.53	0.45–0.71
ABW:IPD						
L1	1.6	0.81	0.50	0.70	0.11	0.49–0.90
L2	1.6	0.95	0.67	0.71	0.09	0.44–0.99
L3	1.6	0.90	0.67	0.77	0.03	0.60–0.95
L4	1.7	0.78	0.83	0.83	0.01	0.72–0.94
L5	1.7	0.61	0.83	0.72	0.09	0.58–0.85
S1	1.7	0.68	0.99	0.83	0.01	0.72–0.94

*Abbreviations*: *SD* standard deviation, *SBW* sagittal vertebral body width, *PW* pedicle width, *PPM* posterior pedicle margin, *ABW* axial vertebral body width, *IPD* interpedicular distance

## Discussion

With these radiographic indices, patients with lumbar DSS can be identified on either the AP or lateral lumbar spine radiographs, which can produce the same diagnostic purpose as MRI. From the results, absolute measurements of PW generally decrease from cranial to caudally in both groups. These measurements mirror that of the AP bony spinal canal diameter and are thus a good representation of the actual MRI findings. SBW and PPM appears to differ between the groups as there is a gradual change in size for the patient group while they stay similar across levels in the control group. In addition, the measurements of the ABW and IPD increase from cranial to caudally in both groups. These findings further support the fact that the AP bony spinal canal diameter (or the PW in this study) is most predictive of DSS since it is likely to be independent from the patient size which is something that cannot be derived from the IPD. Hence, it is likely that the cutoff values provided by the SBW:PW radio is more predictive of DSS. Whether this is true or not requires further investigation.

Previously, there has been no agreement on the clinical or radiological definition of lumbar canal stenosis despite many imaging and cadaveric studies [7, 10, 11, 14–17, 23, 34, 35]. Reasons for these discrepancies are based on the lack of a uniformed method of measurement for the bony spinal canal diameter. DSS can now be defined based on a standardized method for the assessment of spinal canal MRI phenotypes [2]. In this study, patients with DSS are diagnosed by the AP bony spinal canal diameter phenotype on MRI, which is the parameter determined to be the most representative of DSS and can be obtainable from axial MRI images [2, 31]. However, due to the obvious cost-related concerns of MRI, this study is conducted to develop new phenotypes of DSS on radiographs using easily measurable radiographic parameters. In terms of radiation exposure, only two standing radiographs are required for assessment, and these are usually required prior to any treatment to assess the loaded spine since MRIs are performed in supine. Thus, the clinical risk of these radiographs is minimal. Use of scanning systems like the EOS® will require further study to assess feasibility and reliability of measurements.

It is important to note that these indices are created based on a cohort of both symptomatic patients requiring surgical decompression and asymptomatic subjects recruited from the general population. Interestingly, none of the subjects in the control group has DSS on MRI measurements. This suggests that DSS is likely an important parameter that differentiates subjects who become symptomatic requiring surgery and those that may remain asymptomatic. Although this can be theorized from our results, at present, these indices can only serve as reference for identifying subjects with narrowed spinal canals without further longitudinal follow-up of these asymptomatic individuals. These radiographic indices are not meant to be a guide to whether a patient deserves decompression or not. Symptomatology is not a parameter we used to define these indices, and not all developmentally narrowed levels may be symptomatic. The overall denominator of subjects with DSS is unknown in the general population, and thus, what is considered “healthy” or “normal” is unknown without large-scale population studies.

Developmentally, the pedicle is the main reason for a narrowed spinal canal. Applying the knowledge from patients with achondroplasia, a disorder in endochondral ossification leads to fusion of pedicles to vertebral bodies; formation of abnormally short pedicles and narrowed IPD gives rise to inadequate spinal canal sizes and risk of neurological compromise [36]. In the general population, a widening of the IPD is observed from cranial to caudal spinal segments [37, 38]. This finding is echoed by our study results. As radiographic parameters of pedicle sizes and IPD are more consistent in our subjects, our indices are derived from the PW, PPM, and IPD. Although it is impossible to measure the exact width of the pedicle depicted on lateral radiographs, two consistent landmarks (facet joints and posterior vertebral body) are used to help guide us to where the pedicle should be. The exact location of our measurement parameter is of little concern because we only require a consistent parameter that can reflect a short pedicle. This value is then compared to the vertebral body width to create a ratio. The IPD is another consistent landmark since the well-defined pedicle is usually seen clearly on AP radiographs. However, this is likely not as representative as the PW as the pedicle sizes are more directly related to the AP bony spinal canal diameter measured on MRI. This is supported by our study results which proves that SBW:PW is the most significant index that has strong sensitivity and specificity in identifying DSS especially for L3–S1 which are clinically the more commonly affected levels by lumbar spinal stenosis.

Since all ratios have a component of the vertebral body width, the confounding effect of body size and magnification error can be accounted for. One of the key issues with measurement of the vertebral body width is to avoid measuring any osteophytes anterior to the vertebral body. This can be discerned by locating the most vertical tangential line lateral (for AP radiographs) to or anterior (for lateral radiographs) to the vertebral body using adjacent vertebral bodies as a reference. This is important to avoid a false positive result of narrowed canal due to overestimation of the vertebral body width. In addition, these ratios are based on static bony parameters which are unlikely to be subjected to change with posture or movement as compared to other dynamic measurements. Hence, we can expect these ratios to be consistent even on flexion-extension dynamic radiographs.

The vertebral body height and FW have large variability among the radiographs because they are dependent on a neutral view. Any tilt in the view exposes a double endplate contour because there is no longer overlap between the two sides of the endplate (anterior/posterior for AP view; medial/lateral for lateral view). Similar problem can be seen with scoliosis. Readers would have difficultly deciding on which endplate to measure, hence resulting in poorer reliability between the readers. Furthermore, deformities of vertebral body height are well documented and can be due to age-related effects, congenital problems, or osteoporotic fractures [39]. This will lead to age-dependent variations in measurements. Similar problems are observed with the FW measurements.

The limitation of this study is the lack of longitudinal data. Impactful clinical applications cannot be generated at this stage unless longitudinal follow-up of the patient group with DSS shows recurrence of stenosis at nonoperated levels and the control group without DSS shows no development of stenosis symptoms. This is an important follow-up study since our control group is generally younger than our patient group. The lack of age matching and random selection of subjects are also limitations. Nevertheless, the aim of this study is to present clinically useful indices for diagnosis, and the values were based on clearly distinct groups. A potential limitation of our upper level (L1–L2) indices is the lack of patients with upper level stenosis symptoms. Although these are reference indices based on patients and controls, further correlation analysis between symptoms and canal size is required to better understand its relationship in future studies. As the results of our study are based on MRI and X-ray image assessments, at this stage, these radiographic measurements are useful for classifying a subject as having normal or developmentally narrowed spinal canals but they cannot be used for influencing clinical decision and outcomes of surgery. In addition, there is an inherent bias with open recruitment as the possible underlying reason for these “normal” subjects to actively engage us for imaging may be because they experience, however mild, some sort or spinal disorder or symptom.

## Conclusions

To our knowledge, this is the first study to identify easy-to-use radiological indices for DSS. Subject identification can be based on a simple radiograph which, as a screening tool, is more cost-efficient and is more readily available than MRI. The radiographic indices created here are sufficient for case identification since they are based on MRI-diagnosed phenotypes and standardized measurement methods. To understand how a developmentally narrowed spinal canal correlates with symptoms requires further understanding of phenotypic differences between symptomatic and asymptomatic DSS as well as longitudinal follow-up studies to determine any age-related effects on measurement parameters. There is also value in comparing measurements in the loaded and the unloaded spine and in other populations and ethnic groups for validation. Future study should further determine the clinical significance of DSS especially with the risk of symptom recurrence and reoperation.

## Abbreviations

ABW: Axial vertebral body width
AP: Anteroposterior
CI: Confidence interval
DSS: Developmental spinal stenosis
FW: Foraminal width
ICC: Intraclass correlation
IPD: Interpedicular distance
MRI: Magnetic resonance imaging
PPM: Posterior pedicle margin
PW: Pedicle width
ROC: Receiver operating characteristic
SBW: Sagittal vertebral body width
TE: Echo time
TR: Repetition time
